# Genicular Nerve Block Versus Genicular Nerve Ablation for Knee Osteoarthritis: A Systematic Review of Randomized Controlled Trials and Retrospective Studies

**DOI:** 10.7759/cureus.79106

**Published:** 2025-02-16

**Authors:** Ammar Yaser Mohammad Toubasi, Amanda Myles, Paramvir Singh, Zhuo Sun, Anterpreet Dua

**Affiliations:** 1 Anesthesiology and Perioperative Medicine, Augusta University Medical College of Georgia, Augusta, USA; 2 Anesthesiology, Boston Medical Center, Boston, USA; 3 Anesthesiology, Piedmont Athens Regional Center, Athens, USA; 4 Anesthesiology, Augusta University Medical College of Georgia, Augusta, USA; 5 Anesthesiology, Augusta University Medical Center, Augusta, USA

**Keywords:** functional improvement, genicular nerve ablation, genicular nerve block, knee osteoarthritis, pain control

## Abstract

This systematic review aimed to compare the efficacy and safety of genicular nerve ablation and genicular nerve block (GNB) in pain control and functional improvement in knee osteoarthritis (OA) patients using a systematic review of randomized controlled trials (RCTs) and retrospective studies. We searched PubMed, Google Scholar, Cochrane, Science Direct, and Web of Science using specific keywords until April 2023. The primary outcome measures were visual analog scale (VAS) and numerical rating scale (NRS) scores for pain. The secondary outcome measures included functional outcomes assessed by the Western Ontario and McMaster Universities Arthritis Index (WOMAC) score and complications. Four RCTs and two comparative studies met the inclusion criteria. The analysis revealed that both genicular nerve ablation and nerve block effectively reduced pain and improved functionality. Ablation possibly provided more substantial and long-lasting effects than diagnostic blocks. However, the superiority of ablation compared to therapeutic block with steroids is still not conclusive in pain reduction. Functional capacity improvements were comparable between ablation and therapeutic block. Adverse events were minimal and transient.

## Introduction and background

Osteoarthritis (OA), a degenerative joint disease, is the most prevalent type of arthritis in the world and results in chronic pain and disability, leading to massive personal costs and a significant impact on the healthcare system worldwide [1]. Knee OA, the most common form of arthritis in the knee, has a prevalence of up to 46% in some countries, and this disease is expected to increase alongside the rise of obesity and the extension of life expectancies [2]. Regarding the prevalence of knee OA, it is vital to recognize the burden on all aspects of one’s quality of life, including work, fitness, and mental health [3]. For people with advanced knee OA, pain often persists when sitting or lying down. Along with the expected effects of aging, this progresses into pain that can be unbearable or disabling. In the United States, the overall economic burden of knee OA is more than $27 billion annually [4,5].

Conservative treatments, including oral or topical medications, physical therapy, and intra-articular injections, are common first-line treatments for knee pain with OA [6]; however, these conservative treatments may be insufficient and offer limited long-term benefits for pain relief and improved functional capacity [7]. Instead, genicular nerve blocks (GNB) and ablation, which aim to selectively inhibit the sensory nerves supplying the knee joint capsule, thereby relieving knee pain and improving function, are therapies that physicians have rapidly incorporated into practice [8-11], especially when total knee replacement surgery is unwarranted or undesired by the patient due to coexisting diseases or health issues. Blocks could be done with local anesthetic only (diagnostic block) or local anesthetic combined with steroids (therapeutic block). A range of ablation techniques, such as thermal, cooled, pulsed, and neurolytic ablations, have been used to treat the genicular nerve. All are very safe procedures with minimal risks that do not put patients at risk of surgical complications seen during the intraoperative or postoperative periods of knee replacements and complications from anesthesia [12].

In recent years, GNB and ablation have been widely applied in practice as a promising intervention with numerous studies published [13]. However, no level 1 study (meta-analysis or systematic review) compares GNB with ablation. Hence, the differentiation of these two therapies remains to be seen, resulting in ambiguity and undefined steps in the treatment process for knee arthritis. Thus, we conducted a systematic review to evaluate the efficacy in pain control and functional capacity of GNB versus ablation in patients with knee OA to provide an evidence-based overview. Our systematic review of randomized controlled trials (RCTs) and comparative studies addresses our hypothesis that genicular nerve ablation manages pain and improves functional capacity greater than GNB in knee OA. Our study is the first review to compare the outcomes of GNB and genicular nerve ablation in knee arthritis patients.

## Review

Material and methods

This systematic review followed the guidelines of the Preferred Reporting Items for Systematic Reviews and Meta-Analyses (PRISMA) [14]. The primary outcome was to compare knee pain using a visual analog scale (VAS) and numerical rating scale (NRS) scores of genicular nerve ablation and nerve block. The secondary outcomes consisted of functional outcomes using the Western Ontario and McMaster Universities Arthritis Index (WOMAC) score and complications.

Search Strategy

Electronic databases, PubMed, Google Scholar, Cochrane, Science Direct, and Web of Science, were searched using the keywords "Genicular Nerve Block" AND "Genicular Nerve Ablation." Studies were screened by titles and abstracts. Two of the present authors performed a full-text review to determine eligibility. The last search was conducted on October 31, 2024.

Study Selection and Data Extraction

Studies comparing genicular nerve ablation and GNB for knee OA were sought. A GNB is “conventionally performed by local anesthesia alone or in combination with corticosteroid.” Inclusion criteria were RCTs and retrospective cohort studies that investigated pain and functional improvement from the treatment. We excluded cadaveric studies and basic research. Two authors independently performed the search and data extraction from published articles.

Quality Assessment

The risk of bias of each RCT was assessed using the Cochrane risk-of-bias tool for randomized trials (RoB 2) [15] and the Newcastle-Ottawa scale (NOS) [16] for retrospective studies. The RoB tool contains five domains: randomization, adherence to intended treatments, missing outcomes, measurement bias, and reporting bias. The NOS tool includes three domains: selection, comparability, and outcome. Two authors independently assessed each study.

Results

Study Selection

**Figure 1:**
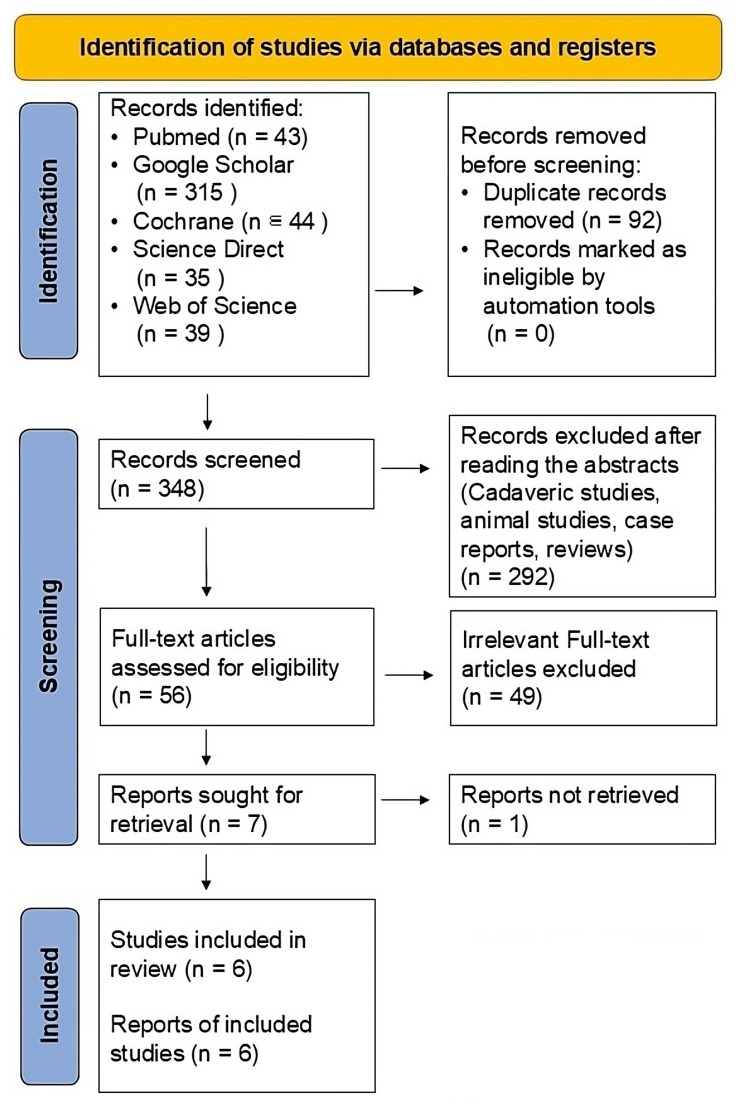
The flow diagram illustrates the results of a literature search comparing GNB and ablation using databases and registries GNB, genicular nerve block

Figure 1 displays the flow diagram illustrating the results of the literature search. Using the search strategy, we identified 476 articles, of which 469 were removed after being reviewed for eligibility. Finally, we included seven eligible studies for full-text reviews. One article was not included because it needed extractable data, resulting in six suitable articles in the synthesis. The qualitative and quantitative analyses included all six eligible studies, as seen in Table 1. 

**Table 1:** Level of evidence and designs of the studies selected RCT, randomized controlled trials

Study number	Author	Year	Level of evidence	Design
1	Malaithong et al. [13]	2022	1	RCT
2	Elashmawy et al. [12]	2022	1	RCT
3	Ghai et al. [14]	2021	1	RCT
4	Carlone et al. [16]	2020	3	Retrospective cohort
5	Kapural et al. [15]	2019	3	Retrospective cohort
6	Choi et al. [5]	2011	1	RCT

Characteristics and Demographics of Included Studies

A summary of the findings of the included studies is presented in Table 2. This synthesis included 619 patients. Of these, 538 patients received GNB, and 367 received genicular nerve ablation. The mean age was 63.6 years, and the mean follow-up was 6.6 months. The main inclusion criteria shared by all studies was chronic knee pain. All studies excluded patients with acute knee pain, prior knee surgery, a recent history of intra-articular knee injection (steroid or hyaluronic acid), or a history of a bleeding disorder, except Elashmawy et al. which included patients with pain after TKA [17]. Detailed information on procedures performed is described in Table 3. For ablation, a variety of settings were applied in selected studies. Among them, the highest temperature used in ablation is 90 degrees Celsius [18], while the lowest one is 42 degrees Celsius [19]. Cooled radiofrequency ablation (RFA) with a generator set temperature of 60 degrees Celsius, resulting in the average maximum tissue temperatures exceeding 80 degrees Celsius was applied in two studies [20,21]. Regarding GNB, bupivacaine and levobupivacaine were the most used agents in these studies [8].

**Table 2:** The main characteristics of the studies included VAS, visual analog scale; OKS, Oxford knee scores; NRS, numerical rating scale; WOMAC, Western Ontario and McMaster Universities Arthritis Index; RFA, radiofrequency ablation; GNB, genicular nerve blocks; CRFA, cooled radiofrequency ablation

Study number	Author	Follow-up (months)	Scale	Main finding
1	Choi et al., 2011 [10]	Baseline, 1 week, 1, 3	VAS, OKS, global perceived effect	VAS scores showed that the RF group had statistically significantly less knee joint pain at 1 month and 3 months compared with the block group. OKS showed similar findings (p<0.001). In the RF group, 59%, 65%, and 59% achieved at least 50% knee pain relief at 1, 4, and 12 weeks, respectively. The VAS scores in the control group were only lower than the baseline in 1st week. The RF group showed superior improvement compared with the control group at both 4 and 12 weeks (P<0.001).
2	Elashmawy et al., 2022 [17]	Baseline, 1, 6	VAS, NRS, WOMAC	VAS, NRS, and WOMAC scores improved significantly after injection in ablation group up to 6 months (p<0.001) while in block group improved for only 1 month.
3	Malaithong et al., 2022 [18]	Baseline, 1, 2, 4, 6, 8, 10, 12	VAS, WOMAC	VAS scores improved significantly for RFA (from 5.7±1.9 to 3.2±2.6) and GNB groups (from 5.0±1.4 to 2.6±2.4), with no significant differences observed between groups at 12-month follow-up (p=0.4) or any other time point. WOMAC scores showed no significant differences (p=0.85) between groups for the secondary outcome and only the control group (GNB) showed significant improvement in function at the 12-month follow-up.
4	Ghai et al., 2021 [19]	Baseline, 1, 2, 3	NRS, WOMAC	NRS scores decreased significantly in both the groups at 12 weeks (NRS decreased by at least 50%: 70% in ablation vs. 66% in block, P>0.99) and other follow-up times compared to baseline. There was also a statistically significant improvement in WOMAC scores in both groups at all follow-up times compared to baseline.
5	Kapural et al., 2019 [20]	Baseline, 12	VAS	The average baseline pain score was 8.5, which decreased to 2.2 after the geniculate local anesthetic block, and to 4.2 after CRFA. A total of 65% of the patients claimed >50% pain relief, whereas 77% had 2 or more VAS points decrease, and 26 (14%) patients reported no pain at all after CRFA. The mean duration of >50% pain relief after CRFA was 12.5 months (range 0-35 months).
6	Carlone et al., 2020 [21]	Baseline, 1-1.5	VAS	In the GNB, 31.8% failed to respond to the procedure. Of the subjects that proceeded to genicular nerve ablation, 53.7% reported less than 50% pain relief, and 46.3% reported pain relief greater than or equal to 50% at the first follow-up visit.

**Table 3:** Ablation settings of selected studies RFA, radiofrequency ablation

Author	Treatment	Temperature setting (°C)	Time (sec)	Guidance
Choi et al., 2011 [10]	RFA	70	90	Fluoroscopic
Elashmawy et al., 2022 [17]	Alcoholic neurolysis	n/a	n/a	Ultrasound
Malaithong et al., 2022 [18]	Bipolar-RFA	90	180	Fluoroscopic
Ghai et al., 2021 [19]	Pulsed RFA	42	120	Ultrasound
Kapural et al., 2019 [20]	Cooled RFA	60	150	Fluoroscopic
Carlone et al., 2020 [21]	Cooled RFA	60	150	n/a

Quality Assessment

**Figure 2:**
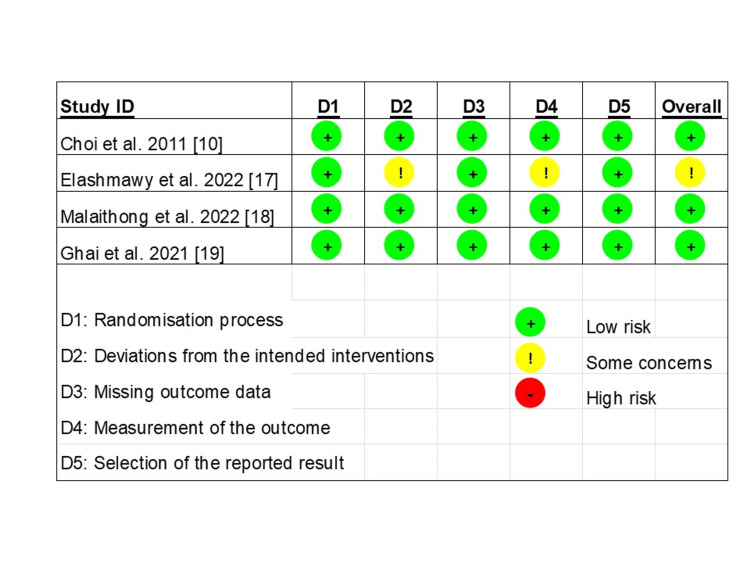
Summary of the risk of bias assessment across the five domains using the Cochrane RoB 2 tool for RCTs

The risk of bias assessment is presented on a scale, as shown in Figure 2. Four RCT studies were evaluated using RoB2 [15], with three studies demonstrating a low risk of bias and one showing some concerns. The NOS [16] was used to assess retrospective studies. Both studies scored four stars in the selection domain and a maximum of two stars in the comparability domain. Regarding the outcome domain, Carlone et al. scored two stars, while Kapural et al. scored three. A summary of the scale is shown in Table 4.

**Table 4:** Qualitative analysis of included retrospective studies

Study	Selection	Comparability	Outcome	Quality score
Kapural et al., 2019 [20]	****	**	***	Good
Carlone et al., 2020 [21]	****	**	**	Fair

Results of individual studies

Pain Scores

The NRS [22] for pain was reported in two out of six studies. Both studies showed significant differences in pain at all time points. Elashmawy et al. observed that VAS improved significantly after ablation for up to six months while the improvement only lasted for one month in the block group. However, Ghai et al. found that both block and ablation provided significant improvement over 12 weeks. 

The VAS, another score used to assess pain levels [23], was documented in six studies. Among these, Malaithong et al. observed significant reductions in pain for both block and ablation groups at 12 months but reported no significant differences in VAS scores between the groups. Elashmawy et al. found a substantial decrease in pain at both one and six months in both groups, though the ablation provided more improvement (with a VAS score of eight) than the effects provided by the block group (with a VAS score of seven). Carlone et al. reported that 68.2% of patients experienced ≥50% pain relief after the initial block, while only 46.3% of those who underwent ablation reported similar pain relief, with a mean improvement of 36.7% at one month. Among the patients who responded to ablation, the mean duration of pain relief greater than 50% following the treatment was 12.5 months. Kapural et al. also found that 84% of patients had ≥50% pain relief after the initial block, and 65% of those who underwent ablation experienced similar pain relief. Finally, Choi et al. reported that the ablation group showed significantly greater pain improvement in VAS than the block group at both one-month and three-month follow-ups.

Functional Scores

The WOMAC score [24] was used in three studies to assess functional improvement following the treatment. Malaithong et al. reported no significant changes in the WOMAC scores between block and ablation groups at any time during the follow-up, except for the control group at the 12-month follow-up. Similarly, Ghai et al. observed that there was a significant improvement from block and ablation compared to baseline, though the difference between these two groups was minimal. In contrast, Elashmawy et al. found significant improvements in both groups at one and six months, with the ablation group showing notably better results compared to the block group. Oxford knee scores (OKS) were applied to evaluate functional changes in Choi’s study. They observed that the RFA group had statistically better OKS scores [25] than the block group at both one- and three-month follow-ups, which is similar to the finding from Elashmawy’s study.

Complications

The post-procedural complications in all included studies are summarized in Table 5.

**Table 5:** Post-procedural complications

Study	Post-procedural complication
Choi et al., 2011 [10]	No adverse events
Elashmawy et al., 2022 [17]	No significant difference between groups in all complications (local pain, hypoesthesia, swelling, and bruise), and nothing persisted beyond two weeks
Malaithong et al., 2022 [18]	Procedural complications including hematoma, infection, and neurological complications (e.g., abnormal proprioception, numbness, paresthesia, neuralgia, and motor weakness) were recorded per authors but not included in the study
Ghai et al., 2021 [19]	No adverse events
Kapural et al., 2019 [20]	No adverse events
Carlone et al., 2020 [21]	No adverse events

Four studies reported no adverse events in both groups. Malaithong mentioned that adverse events were recorded, but these were not included in the study with any comparison between groups. Local pain is the most common complication reported in the studies. Other reported complications include bruises, swelling, and hypoesthesia. All reported complications are transient and last less than two weeks. Both groups have no significant difference regarding complications [17].

Discussion

Numerous articles have shown that blocks and ablations improve pain control and functional capacity, and there is a commonly held perception in the clinical setting that ablations are superior [9,10]. Still, a systematic review has yet to compare both treatments. This review addresses our hypothesis of determining whether genicular nerve ablation reduces pain and improves functional capacity more than GNB in patients with knee OA.

Key Findings

Recently, Manchikanti et al. initiated a study to assess the efficacy and cost-effective analysis of the management of chronic cervical spondylosis pain by using cervical therapeutic medial branch blocks (MBB) versus nerve ablation. The authors found that compared to ablation, the cervical MBB provided comparable clinical outcomes in pain control with similar costs during one-year follow-ups [26]. Another study was conducted on patients with chronic lumbar facet joint pain who underwent either lumbar MBBk or nerve ablation [27]. This study shows identical outcomes of therapeutic lumbar facet joint nerve blocks compared to radiofrequency neurotomy, as indicated by significant pain relief and cost-utility. How about GNB versus ablation? The results and evidence synthesis from the eight studies that met our search criteria show that both methods effectively decrease pain and improve functionality. The genicular nerve ablation is shown to be superior to diagnostic GNB with local anesthetics only when assessing pain at various follow-up times. Choi et al. observed a reduction in pain in the RF group compared with the control group, which was shown on the VAS after four and 12 weeks, and a decrease in knee pain of at least 50% after one, four, and 12 weeks in the RF group. Some practitioners prefer diagnostic blocks with local anesthetic alone, followed by ablation in clinics, while others prefer nerve blocks with steroids. Among the five studies comparing ablation with steroid-based nerve blocks, three observed similar outcomes: both treatments provided comparable pain relief, while two studies found that ablation offered longer-lasting pain relief. Additionally, ablation is generally equivalent to, and in some cases superior to, nerve blocks in terms of improving functional capacity.

One retrospective chart review observed that only 35% of patients reported ≥50% pain reduction from the RFA after the successful diagnostic block, and the author believes that it is crucial to identify predictors of pain reduction before moving to genicular RFA [28].

Concerning pain scores using both NRS and VAS, significant differences were found in the studies included at various time points, and they all showed significant reduction in pain for ablation greater than that for block. Two of the studies showed that for patients who initially had blocks, significant percentages (46.3% and 84%) had further increased pain reduction with the addition of ablation versus those without. In reviewing the functional scores (WOMAC, Knee Society Score (KSS), OKS), which were included in four of the six studies, significant improvements were noted for both block and ablation groups at follow-up times without statistical difference for three of the studies, and one of these studies reported notable improvement in ablation group compared to the block group. One of the studies showed no significant improvement in functional WOMAC scores except in the control GNB group at 12-month follow-up. Regarding post-procedural complications, the studies either did not include difficulties, reported no problems, or it was shown that there was no significant difference between both groups in all complications.

Based on current studies, we found that ablation exhibited a more substantial and long-lasting analgesic effect than diagnostic blocks. However, the efficacy between the therapeutic block and ablation is still inconclusive. We also did not find a published study to compare the cost-utility between therapeutic block and ablation. We also did not find a published study to compare the cost-utility between genicular nerve therapeutic block and ablation. Based on the current review, we cannot recommend one treatment option over the other. However, considering the duration, the stress and pain most patients experienced during the treatment, and the cost of nerve ablation, it is reasonable to recommend that nerve blocks with steroids be considered before moving to ablation.

Strength and Limitation

This review's strengths include a higher level of evidence, including only RCTs and comparative studies. Additionally, most of the studies included in the review have been published in the last five years. The six studies share similar outcomes, allowing for a simple, clearly defined result comparison.

The main limitation of our review is the small sample size due to the limited number of studies currently available that address our research question. Given the limited data and analyses, more high-level evidence is needed to perform a robust meta-analysis. Additionally, more studies are required to include detailed methodological analyses, such as comparing different types of ablations (radiofrequency, cooled, and chemical) and various image-guided modalities (ultrasound-guided versus fluoroscopy-guided). Regarding the efficacy of other ablation techniques, Chou et al. conducted a meta-analysis and found no significant differences in pain relief among conventional, pulsed, and cooled ablations [29]. GNB and ablations were initially performed using fluoroscopic guidance. Recently, ultrasound-guided GNB and ablation have been introduced into practice. Ultrasound guidance not only reduces radiation exposure but also allows for visualization of the genicular arteries and, in some cases, the genicular nerves. Huang et al. demonstrated that block and ablation performed under ultrasound guidance is a safe and effective alternative [30]. Kim et al. performed a prospective randomized study comparing the efficacy of ultrasound- versus fluoroscopy-guided GNB for chronic OA and found no significant difference in pain relief, functional improvement, or safety between the two groups [31]. Identifying precise anatomical landmarks is crucial for successful GNB or ablation. Fonkoue et al. introduced a new technique that, in addition to blocking the three conventional nerve branches (superomedial, superolateral, and inferomedial genicular nerves), also blocks the recurrent fibular nerve and infrapatellar branch of the saphenous nerve [32]. This revised approach led to greater pain relief and a higher proportion of successful responders one hour post-intervention. Some studies have even performed blocks at up to 10 locations to improve the treatment of more sensory nerves around the knee and address anatomical variations [33,34]. In addition to the technologies, the medications and dosages used in GNB and ablation vary across studies [35,36]. All these factors may contribute to differences in efficacy among studies.

Another limitation of our review is the absence of demographic sub-criteria, such as OA grade, whether a total knee replacement was performed prior to treatment, and the targeted compartment of the genicular nerve. There is considerable heterogeneity among study protocols, including differences in techniques (e.g., ablation temperatures, nerve targets, imaging modalities), as well as variations in outcome reporting, which make further evaluation challenging. Additionally, limited follow-up durations create uncertainty regarding long-term efficacy.

Future Research Direction

Further clinical studies are needed to address these knowledge gaps, including evaluating the efficacy of specific ablation techniques and image-guided modalities, as well as assessing clinical outcomes relevant to more defined demographic and pathological variables.

## Conclusions

In summary, ablation and blocking of the genicular nerve significantly reduced pain and improved WOMAC scores in patients with OA knee pain without significant complication. Ablation possibly exhibited a more substantial and long-lasting analgesic effect than blocks. However, the efficacy between therapeutic block and ablation is still not conclusive based on current studies as there are not enough high-quality studies for further evaluation. We also did not find a published study to compare the cost-utility between therapeutic block and ablation. Further studies addressing the clinical outcomes and cost-effectiveness of large prospective multicenter RCTs are needed to direct clinical practice. 
